# Assessment of the modulation degrees of intensity-modulated radiation therapy plans

**DOI:** 10.1186/s13014-018-1193-9

**Published:** 2018-12-13

**Authors:** So-Yeon Park, Jung-in Kim, Minsoo Chun, Hyunjun Ahn, Jong Min Park

**Affiliations:** 1 Department of Radiation Oncology, Veterans Health Service Medical Center, Seoul, Republic of Korea; 2 Institute of Radiation Medicine, Seoul National University Medical Research Center, Seoul, Republic of Korea; 3 Department of Radiation Oncology, Seoul National University Hospital, Seoul, Republic of Korea; 4 Biomedical Research Institute, Seoul National University College of Medicine, Seoul, Republic of Korea; 5 Institute for Smart System, Robotics Research Laboratory for Extreme Environments, Advanced Institutes of Convergence Technology, Suwon, Republic of Korea

**Keywords:** Modulation index, Intensity-modulated radiation therapy, Plan delivery accuracy

## Abstract

**Background:**

To evaluate the modulation indices (MIs) for predicting the plan delivery accuracies of intensity-modulated radiation therapy (IMRT) plans.

**Methods:**

A total of 100 dynamic IMRT plans that used TrueBeam STx and 102 dynamic IMRT plans that used Trilogy were selected. For each plan, various MIs were calculated, which included the modulation complexity score (MCS), plan-averaged beam area (PA), plan-averaged beam irregularity (PI), plan-averaged beam modulation (PM), MI quantifying multi-leaf collimator (MLC) speeds (MI_s_), MI quantifying MLC acceleration (MI_a_), and MI quantifying MLC acceleration and segment aperture irregularity (MI_c,IMRT_). To determine plan delivery accuracy, global gamma passing rates, MLC errors of log files, and dose-volumetric parameter differences between original and log file-reconstructed IMRT plans were obtained. To assess the ability of each MI for predicting plan delivery accuracy, Spearman’s rank correlation coefficients (*r*_*s*_) between MIs and plan delivery accuracy measures were calculated.

**Results:**

PI showed moderately strong correlations with gamma passing rates in MapCHECK2 measurements of both TrueBeam STx and Trilogy (*r*_*s*_ = − 0.591 with *p* <  0.001 and − 0.427 with *p* <  0.001 to with gamma criterion of 2%/2 mm, respectively). For ArcCHECK measurements, PI also showed moderately strong correlations with the gamma passing rates in the ArcCHECK measurements of TrueBeam STx and Trilogy (*r*_*s*_ = − 0.545 with *p* <  0.001 and *r*_*s*_ = − 0.581 with *p* <  0.001 with gamma criterion of 2%/2 mm, respectively). The PI showed the second strongest correlation with MLC errors in both TrueBeam STx and Trilogy (*r*_*s*_ = 0.861 with *p* <  0.001 and *r*_*s*_ = 0.767 with *p* <  0.001, respectively). In general, the PI showed moderately strong correlations with every plan delivery accuracy measure.

**Conclusions:**

The PI showed moderately strong correlations with every plan delivery accuracy measure and therefore is a useful predictor of IMRT delivery accuracy.

**Electronic supplementary material:**

The online version of this article (10.1186/s13014-018-1193-9) contains supplementary material, which is available to authorized users.

## Background

Intensity-modulated radiation therapy (IMRT) delivers highly conformal radiation dose distributions to target volumes, while sparing normal tissues [1–8]. As the IMRT plan complexity increases to acquire the desired dose distribution, i.e., the modulation degree of IMRT increases, a large number of small and irregularly shaped beam apertures are utilized. It results in increased dose calculation and multi-leaf collimator (MLC) movement uncertainties [4, 9–11]. Therefore, the probability that the intended dose distribution is not accurately delivered to the patient during treatment increases. In this respect, verification of IMRT plan delivery accuracy is highly recommended before treatment in a clinical setting.

To verify IMRT plan delivery accuracy, gamma evaluations have been widely adopted in the clinic, as pre-treatment quality assurance (QA) methods [12–14]. Although gamma evaluation is a practical method, it has some limitations. It has been reported that the gamma passing rates of individual dosimeters have been inconsistent with respect to each other [15]. Therefore, a thorough gamma evaluation validation of each device that is installed in the clinic should be performed. Moreover, recent studies have demonstrated the clinical irrelevance of the gamma passing rates [16–18]. As an alternative to the gamma evaluation, an analysis of the machine log files that are recorded during delivery has been recommended as a pre-treatment QA by several studies [19–22]. This method can be expanded to analyze the differences between dose-volume parameters of the original plan and the plans that are reconstructed with machine log files [23–25]. However, this method has limitations, as it does not provide an independent verification of linac beam delivery.

On the other hand, to predict IMRT delivery accuracy and calculate the complexity of IMRT plans, several studies have introduced modulation indices (MIs) [4, 10, 26, 27]. Webb et al. measured the modulation degree of IMRT plans by quantifying the changes of adjacent beamlets that exceeded a pre-defined tolerance levels, derived with standard deviations of the fluence map [10]. McNiven et al. designed modulation complexity score (MCS) quantifications of beam aperture and MLC position variations. The MCSs were found to vary from 0 to 1 and decreased as the IMRT plan modulation degrees increased [27]. Du et al. quantified the complexities of both the IMRT and volumetric modulated arc therapy (VMAT) plans, using the beam aperture areas and perimeters, to quantify the usage of small and irregularly shaped beam apertures [4]. For VMAT, Park et al. expanded the concept of Webb’s MI to comprehensively assess MLC speeds and accelerations, the variation of gantry rotation speed, the variation of dose-rate, and the usage of small and irregularly shaped beam apertures [26].

Previous studies have investigated the performance of several MIs for predicting IMRT delivery accuracy, through the use of correlation analyses on conventional plan delivery accuracy measures, such as gamma passing rates. However, there are limitations to evaluating MIs with a single type of linac, single photon energy, limited treatment sites, or using a single type of measuring device. Therefore, further studies need to evaluate the performance of various MIs that are used as clinical predictors of IMRT plan deliveries. In this study, we assessed the performances of various MIs that have been suggested by previous studies to predict IMRT plan delivery accuracy with correlation analyses.

## Methods

### Patient selection and IMRT planning

Two machines, Trilogy and TrueBeam STx (Varian Medical Systems, Palo Alto, CA), which have different plan delivery systems and machine log file generation systems, were selected for this study. After approval by the institutional review board (IRB), a total of 181 patients with various treatment sites were retrospectively selected, and a total of 202 dynamic IMRT plans were generated for this study.

With the TrueBeam STx system, a total of 100 dynamic IMRT plans were generated for head and neck (HN) cancer (20 cases), brain tumor (20 cases), liver cancer (20 cases), spine tumor (20 cases), and lung cancer (20 cases). For HN cancer, a simultaneous integrated boost (SIB) technique was applied. For liver cancer, spine tumors, and lung cancer, stereotactic ablative radiotherapy (SABR) was performed using flattening filter free (FFF) photon beams of 6 MV (6 FFF) and 10 FFF. With Trilogy, a total of 102 dynamic IMRT plans were generated for HN cancer (40 cases), prostate cancer (21 primary plans and 21 boost plans), liver cancer (11 cases), and spine tumor (9 cases). The HN IMRT plans were generated with the SIB technique as with TrueBeam STx system. Information of IMRT plans generated in this study is summarized in Additional file 1: Table S1.

Every IMRT plan in this study was generated in Eclipse™ (Varian Medical Systems, Palo Alto, CA). All IMRT plans used in this study were optimized with the dose volume optimizer (DVO, ver. 10, Varian Medical Systems, Palo Alto, CA), and the dose distributions were calculated using the anisotropic analytic algorithm (AAA, ver. 10, Varian Medical Systems, Palo Alto, CA), with a calculation grid of 1 mm. All IMRT plans were normalized to cover 100% of the target volumes with 90% of the prescription doses.

### Calculation of IMRT plan modulation indices

For each IMRT plan, the MCS from McNiven et al.; the plan-averaged beam area (PA), plan-averaged beam irregularity (PI), and plan-averaged beam modulation (PM) from Du et al.; the modulation index quantifying the speed of MLC (MI_s_); the modulation index for quantifying the MLC acceleration (MI_a_); and the modulation index for quantifying the MLC acceleration and aperture irregularity of the IMRT plan (MI_c,IMRT_) were calculated. Although MI_c,IMRT_ values were originally designed for VMAT, we modified these indices to consider only MLC movements for application to IMRT plans. In other words, we excluded gantry rotation accuracy and dose-rate variability evaluations from the MI_c,IMRT_ value. To eliminate the influence of various segment numbers on the MCS, MI_s_, MI_a_, and MI_c,IMRT_ values, we normalized the values of these MIs with the total segment numbers. Equations of the MIs that were calculated in this study (including modifications) are summarized in Additional file 1: Table S2.

### Measures of IMRT plan delivery accuracy

To evaluate plan delivery accuracy, three types of IMRT plan verification methods were used in this study, which included the global gamma evaluation with absolute doses, differences in MLC positions between planned and the actual deliveries utilizing machine log files, and the differences in the dose-volumetric parameters that were calculated from the original IMRT plans and from the IMRT plans that were reconstructed with machine log files.

For global gamma evaluation, reference dose distributions were calculated in the Eclipse system, with a calculation grid size of 1 mm. The calculated dose distributions were compared to the measured dose distributions using MapCHECK2™ and ArcCHECK™ detector arrays (Sun Nuclear Corporation, Melbourne, FL). Global gamma evaluations with gamma criteria of 3%/3 mm, 2%/2 mm, 2%/1 mm, and 1%/2 mm were performed with absolute doses. Percent dose differences in the global gamma evaluation were calculated relative to the maximum dose in the calculated dose distribution. Before the gamma evaluation measurements, MapCHECK2 and ArcCHECK dosimeters were calibrated, according to manufacturer guidelines.

When IMRT plans were delivered to the dosimeters for gamma evaluation, the actual MLC positions and delivered monitor units (MUs) at each segment were obtained from the DynaLog (Trilogy) and Trajectory (TrueBeam STx) files. These log files were reconstructed as DICOM-RT files, using an in-house program that was written in MATLAB (R2016a, Mathworks Inc., Natick, MA). The differences in the MLC positions between the original IMRT plan and the actual delivery were calculated and averaged for each IMRT plan. After the reconstructed DICOM-RT files from the log files were imported in the Eclipse system, dose distributions were calculated with the same dose calculation condition of the original IMRT plan. With the dose distributions calculated from reconstructed IMRT plans, dose-volumetric parameters for the target volumes and organs at risk (OARs) were calculated and compared to those of the original IMRT plans. The total number of analyzed dose volumetric parameters of TrueBeam STx and Trilogy were 156 and 152, respectively.

### Correlation analysis

To evaluate the performance of each MI that was calculated in this study as an indicator of IMRT delivery accuracy, Spearman’s rho (*r*_*s*_) and corresponding *p* values were calculated between the MIs and the IMRT delivery accuracy measures. In the correlation analysis of different dose-volumetric parameters, the sample sizes were different from one another and the numbers of analyzed dose-volumetric parameters were large. For these reasons, we only counted the number of *r*_*s*_ values with corresponding *p* values less than 0.05, which were considered as statistically significant in this study. The correlations with *r*_*s*_ values from 0.2 to 0.39 are regarded as weak correlations. Those from 0.4 to 0.59 and 0.6 to 1 are regarded as moderately strong and very strong correlations, respectively [27, 28].

## Results

### The values of modulation indices

The values of MIs are shown in Table 1. The values of MIs showed various tendencies, depending on the type of linac and treatment sites.

**Table 1:** The average values of various modulation indices for intensity modulated radiation therapy plans

	MI_s_ (× 10^− 3^)	MI_a_ (× 10^− 2^)	MI_c, IMRT_ (× 10^− 1^)	MCS (× 10^− 1^)	PA (× 10^− 1^)	PI (× 10^− 1^)	PM
TrueBeam STx							
Lung SABR (*n* = 20)	7.97 ± 1.41	2.67 ± 0.69	0.31 ± 0.08	1.25 ± 0.18	1.01 ± 0.45	0.73 ± 0.18	0.67 ± 0.05
Spine SABR (*n* = 20)	5.62 ± 1.48	1.46 ± 0.48	0.17 ± 0.05	0.64 ± 0.19	1.13 ± 0.53	1.53 ± 1.35	0.86 ± 0.05
Liver SABR (*n* = 20)	7.83 ± 2.46	2.40 ± 0.60	0.27 ± 0.06	1.11 ± 0.42	1.39 ± 0.67	1.03 ± 0.65	0.73 ± 0.12
Brain (*n* = 20)	9.20 ± 2.56	9.06 ± 2.62	1.01 ± 0.28	1.40 ± 0.46	3.72 ± 1.85	0.70 ± 0.27	0.67 ± 0.13
Head and neck (*n* = 20)	6.12 ± 2.24	7.06 ± 1.52	0.82 ± 0.17	0.35 ± 0.08	3.72 ± 0.82	2.30 ± 0.43	0.93 ± 0.02
Trilogy							
Head and neck (*n* = 40)	4.14 ± 1.11	5.94 ± 0.83	0.70 ± 0.10	0.30 ± 0.05	2.27 ± 0.41	1.87 ± 0.22	0.94 ± 0.01
Prostate primary (*n* = 21)	3.34 ± 0.71	4.95 ± 0.79	0.58 ± 0.09	0.81 ± 0.14	0.95 ± 0.22	0.64 ± 0.11	0.79 ± 0.04
Prostate boost (*n* = 21)	3.18 ± 0.43	4.89 ± 0.65	0.57 ± 0.07	0.94 ± 0.13	0.90 ± 0.14	0.47 ± 0.10	0.72 ± 0.04
Liver (*n* = 11)	5.14 ± 2.14	6.93 ± 1.40	0.80 ± 0.16	0.78 ± 0.24	1.72 ± 0.60	0.94 ± 0.54	0.79 ± 0.07
Spine (*n* = 9)	5.46 ± 2.68	4.02 ± 1.55	0.47 ± 0.17	0.63 ± 0.25	2.01 ± 1.40	1.26 ± 0.82	0.86 ± 0.06

Note: *MI*_*s*_ Modulation index considering multi-leaf collimator speed, *MI*_*a*_ Modulation index considering multi-leaf collimator acceleration, *MI*_*c, IMRT*_ Modulation index considering multi-leaf collimator acceleration and field aperture irregularity for intensity modulated radiation therapy, *MCS* Modulation complexity score, *PA* Plan-averaged beam area, *PI* Plan-averaged beam irregularity, *PM* Plan-averaged beam modulation, *SABR* Stereotactic ablative radiotherapy, *Prostate primary* Prostate primary plan, *Prostate boost* Prostate boost plan

### Measurement of plan delivery accuracies

The global gamma passing rates with gamma criteria of 3%/3 mm, 2%/2 mm, 2%/1 mm, and 1%/2 mm are shown in Table 2. The mean gamma passing rate for each treatment site was remarkably different from one another at gamma criteria of 2%/1 mm and 1%/2 mm.

**Table 2:** Average values of global gamma passing rates of intensity modulated radiation therapy plans

	3%/3 mm			2%/2 mm			2%/1 mm			1%/2 mm		
	MC	AC	*p*	MC	AC	*p*	MC	AC	*p*	MC	AC	*p*
TrueBeam												
Lung SABR (*n* = 2)	98.5 ± 2.3	99.4 ± 0.6	< 0.001	93.8 ± 4.0	97.7 ± 1.7	< 0.001	85.3 ± 7.1	91.1 ± 3.6	< 0.001	75.2 ± 11.7	95.9 ± 2.7	< 0.001
Spine SABR (*n* = 20)	98.4 ± 2.7	99.3 ± 0.9	< 0.001	95.8 ± 4.1	97.9 ± 1.5	< 0.001	91.0 ± 7.1	87.6 ± 4.3	0.047	91.1 ± 4.3	95.6 ± 2.6	< 0.001
Liver SABR (*n* = 20)	99.2 ± 0.9	99.4 ± 1.2	< 0.001	96.7 ± 2.4	97.9 ± 3.1	< 0.001	92.7 ± 4.0	88.8 ± 7.3	0.013	91.1 ± 5.3	95.8 ± 4.7	< 0.001
Brain (*n* = 20)	98.8 ± 1.6	99.3 ± 2.4	0.002	96.4 ± 3.3	98.5 ± 4.2	0.002	91.6 ± 4.7	92.1 ± 7.3	0.761	89.8 ± 7.1	97.2 ± 4.9	< 0.001
Head and neck (*n* = 20)	96.2 ± 3.4	97.5 ± 3.6	0.072	84.8 ± 8.3	89.1 ± 10.3	0.107	74.5 ± 10.8	80.5 ± 13.9	0.996	66.3 ± 9.9	71.6 ± 13.0	0.001
Trilogy												
Head and neck (*n* = 40)	99.4 ± 0.6	99.8 ± 0.7	0.088	96.3 ± 2.4	98.5 ± 2.1	< 0.001	90.1 ± 3.9	94.4 ± 3.7	0.003	87.8 ± 5.3	94.8 ± 3.9	< 0.001
Prostate primary (*n* = 21)	98.2 ± 0.8	99.5 ± 0.4	0.162	93.9 ± 1.4	97.1 ± 1.0	0.042	84.8 ± 3.0	86.1 ± 2.7	0.077	85.4 ± 1.7	93.4 ± 1.8	< 0.001
Prostate boost (*n* = 21)	97.3 ± 1.5	99.4 ± 0.5	0.624	89.4 ± 2.6	96.2 ± 1.9	0.187	78.9 ± 5.0	83.6 ± 4.9	0.043	73.1 ± 4.9	92.5 ± 2.9	0.005
Liver (*n* = 11)	99.2 ± 0.8	99.8 ± 0.2	0.418	97.3 ± 2.6	98.2 ± 1.4	0.083	93.9 ± 3.9	92.3 ± 4.2	0.814	90.4 ± 4.1	94.5 ± 2.9	< 0.001
Spine (*n* = 9)	99.2 ± 0.9	99.7 ± 0.4	0.246	96.4 ± 3.4	97.3 ± 1.6	0.152	91.5 ± 6.6	89.3 ± 6.8	0.135	91.0 ± 5.5	92.5 ± 2.5	0.153

Note: *MC* MapCHECK2™ detector array, *AC* ArcCHECK™ detector array, *SABR* Stereotactic ablative radiotherapy, *Prostate primary* Prostate primary plan, *Prostate boost* Prostate boost plan

**Fig. 1:**
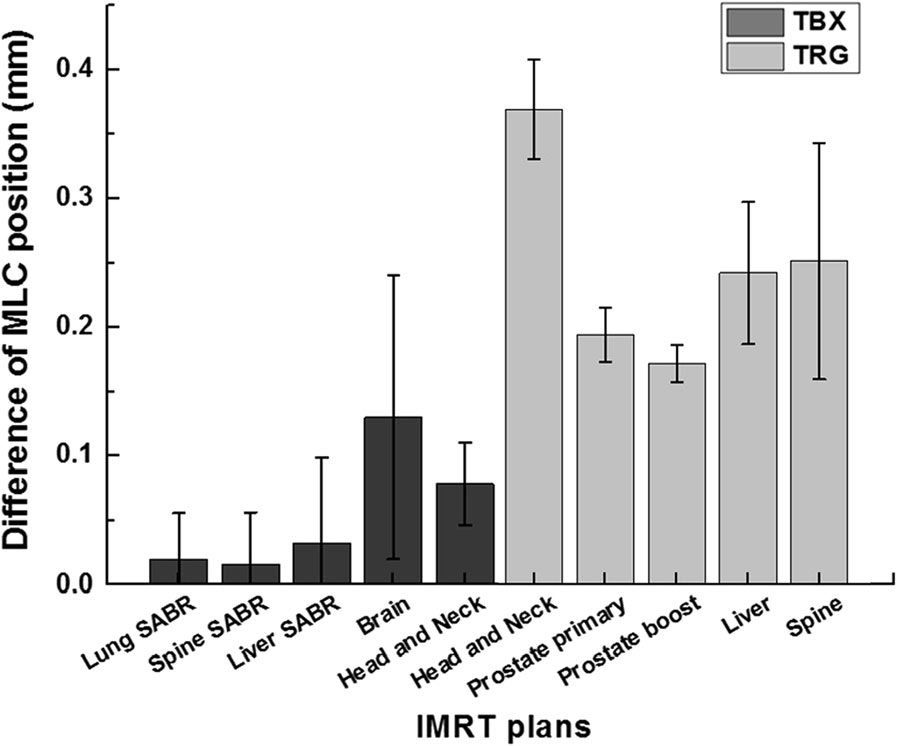
Average and standard deviation of differences in MLC positions between the treatment plans and log files are shown. Dark gray bars represent intensity-modulated radiation therapy (IMRT) plans of TrueBeam STx (TBX) and light gray bars represent IMRT plans of Trilogy (TRG)

The differences between the planned and actual MLC positions are shown in Fig. 1. The average MLC differences of TrueBeam STx and Trilogy were 0.06 mm and 0.25 mm, respectively. For TrueBeam STx, the average MLC difference of the brain IMRT plans was the highest, at 0.13 mm, while for Trilogy, the average MLC difference of the HN IMRT plans was the highest (0.37 mm). The MLC errors of TrueBeam STx were much smaller than those of the Trilogy system.

#### Correlations between gamma passing rates and modulation indices

The *r*_*s*_ values of MIs for the global gamma passing rates, with gamma criteria of 3%/3 mm, 2%/2 mm, 2%/1 mm and 1%/2 mm, of the TrueBeam STx and Trilogy systems are shown in Tables 3 and 4, respectively. The results presented the *r*_*s*_ values with statistically significant *p* values less than 0.05.

**Table 3:** Correlations between global gamma passing rates with various gamma criteria and the modulation indices for intensity modulated radiation therapy plans of TrueBeam STx (*p* ≤ 0.05)

	3%/3 mm		2%/2 mm		2%/1 mm		1%/2 mm	
	*r*_s_	*p*	*r*_s_	*p*	*r*_s_	*p*	*r*_s_	*p*
MapCHECK2								
MI_s_	− 0.241	0.016	− 0.200	0.046	–	–	–	–
MI_a_	–	–	–	–	–	–	–	–
MI_c, IMRT_	–	–	–	–	–	–	− 0.201	0.045
MCS	0.453	< 0.001	0.548	< 0.001	0.509	< 0.001	0.460	< 0.001
PA	−0.223	0.026	− 0.236	0.018	–	–	− 0.196	0.050
PI	−0.465	< 0.001	−0.591	< 0.001	− 0.550	< 0.001	− 0.474	< 0.001
PM	− 0.476	< 0.001	− 0.566	< 0.001	− 0.516	< 0.001	− 0.438	< 0.001
ArcCHECK								
MI_s_	− 0.375	< 0.001	− 0.341	0.001	−0.518	< 0.001	− 0.292	0.003
MI_a_	−0.234	0.019	–	–	− 0.234	0.019	–	–
MI_c, IMRT_	−0.225	0.025	–	–	−0.228	0.022	–	–
MCS	0.431	< 0.001	0.593	< 0.001	0.451	< 0.001	0.699	< 0.001
PA	−0.235	0.019	–	–	− 0.225	0.024	–	–
PI	−0.340	0.001	−0.545	< 0.001	−0.328	0.001	−0.677	< 0.001
PM	−0.369	< 0.001	−0.545	< 0.001	− 0.389	< 0.001	− 0.667	< 0.001

Note: *r*_*s*_ Spearman’s rho, *MI*_*s*_ Modulation index considering multi-leaf collimator speed, *MI*_*a*_ Modulation index considering multi-leaf collimator acceleration, *MI*_*c, IMRT*_ Modulation index considering multi-leaf collimator acceleration and field aperture irregularity for intensity modulated radiation therapy, *MCS* Modulation complexity score, *PA* Plan-averaged beam area, *PI* Plan-averaged beam irregularity, *PM* Plan-averaged beam modulation

**Table 4:** Correlations between global gamma passing rates with various gamma criteria and the modulation indices for intensity modulated radiation therapy plans of Trilogy (*p* ≤ 0.05)

	3%/3 mm		2%/2 mm		2%/1 mm		1%/2 mm	
	*r*_s_	*p*	*r*_s_	*p*	*r*_s_	*p*	*r*_s_	*p*
MI	MapCHECK2							
MI_s_	−0.398	< 0.001	−0.386	< 0.001	− 0.347	< 0.001	− 0.323	0.001
MI_a_	−0.302	0.002	−0.305	0.002	−0.397	< 0.001	− 0.210	0.034
MI_c, IMRT_	−0.315	< 0.001	−0.320	< 0.001	− 0.400	< 0.001	− 0.230	0.006
MCS	0.391	< 0.001	0.347	< 0.001	0.377	< 0.001	0.272	0.004
PA	−0.482	< 0.001	− 0.428	< 0.001	−0.655	< 0.001	− 0.286	< 0.001
PI	−0.452	< 0.001	− 0.427	< 0.001	− 0.475	< 0.001	− 0.348	< 0.001
PM	−0.431	0.001	−0.420	0.001	−0.455	< 0.001	− 0.357	0.020
ArcCHECK								
MI_s_	−0.271	0.006	−0.346	< 0.001	−0.355	< 0.001	–	–
MI_a_	−0.364	< 0.001	−0.455	< 0.001	− 0.456	< 0.001	−0.273	0.006
MI_c, IMRT_	−0.375	< 0.001	−0.471	< 0.001	− 0.472	< 0.001	−0.281	0.004
MCS	0.426	< 0.001	0.589	< 0.001	0.660	< 0.001	–	–
PA	−0.505	< 0.001	− 0.667	< 0.001	−0.739	< 0.001	− 0.370	< 0.001
PI	−0.395	< 0.001	− 0.581	< 0.001	−0.663	< 0.001	–	–
PM	−0.410	< 0.001	−0.607	< 0.001	− 0.671	< 0.001	−0.198	0.046

Note: *r*_s_ Spearman’s rho, *MI*_*s*_ Modulation index considering multi-leaf collimator speed, *MI*_*a*_ Modulation index considering multi-leaf collimator acceleration, *MI*_*c, IMRT*_ Modulation index considering multi-leaf collimator acceleration and field aperture irregularity for intensity modulated radiation therapy, *MCS* Modulation complexity score, *PA* Plan-averaged beam area, *PI* Plan-averaged beam irregularity, *PM* Plan-averaged beam modulation

### Correlations between differences in machine parameters and modulation indices

The correlations between MLC errors and various MIs are shown in Table 5. For TrueBeam STx, every MI showed statistically significant correlations with MLC errors, except for MCS and PM. For Trilogy, every MI showed statistically significant correlations with MLC errors. The PI value had *r*_*s*_ values of 0.861 and 0.767 in both the TrueBeam STx and Trilogy systems, respectively, showing the second strongest correlation (all with *p* <  0.001).

**Table 5:** Correlations of the averaged multi-leaf collimator positional errors to the modulation indices (*p* ≤ 0.05)

	TrueBeam STx		Trilogy	
	*r*_s_	*p*	*r*_s_	*p*
MI_s_	0.411	< 0.001	0.213	0.032
MI_a_	0.783	< 0.001	0.489	< 0.001
MI_c, IMRT_	0.887	< 0.001	0.504	< 0.001
MCS	–	–	−0.727	< 0.001
PA	0.841	< 0.001	0.909	< 0.001
PI	0.861	< 0.001	0.767	< 0.001
PM	–	–	0.751	< 0.001

Note: *r*_s_ Spearman’s rho, *MI*_*s*_ Modulation index considering multi-leaf collimator speed, *MI*_*a*_ Modulation index considering multi-leaf collimator acceleration, *MI*_*c, IMRT*_ Modulation index considering multi-leaf collimator acceleration and field aperture irregularity for intensity modulated radiation therapy, *MCS* Modulation complexity score, *PA* Plan-averaged beam area, *PI* Plan-averaged beam irregularity, *PM* Plan-averaged beam modulation

### Correlations between differences in dose-volume parameters and modulation indices

**Fig. 2:**
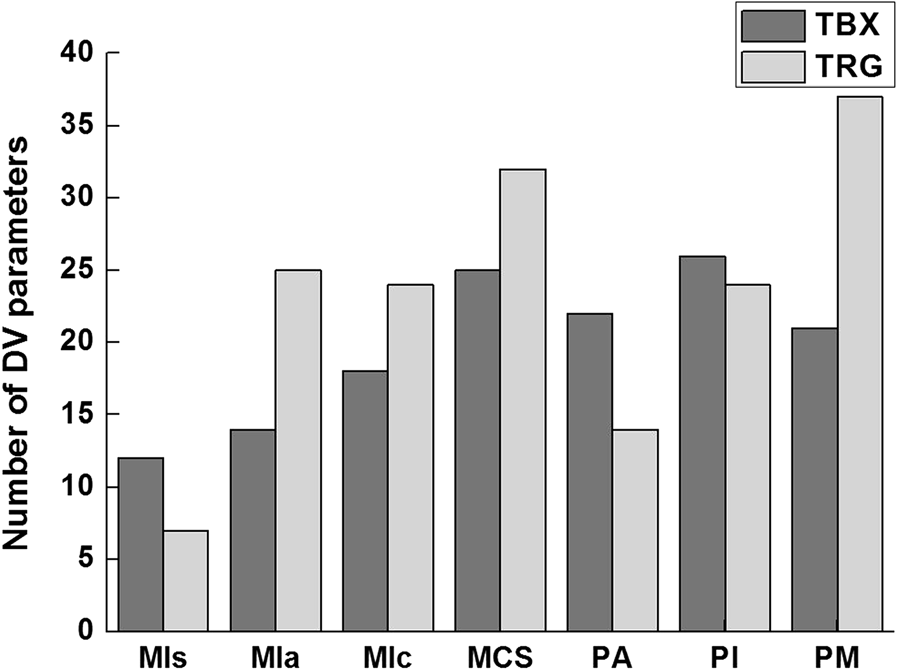
Numbers of statistically significant *r*_*s*_ values (*p* <  0.05) of various MIs to the differences in dose-volumetric parameters between original intensity-modulated radiation therapy (IMRT) plans and the IMRT plans reconstructed with log files are shown. Dark gray bars represent TrueBeam STx (TBX) and light gray bars represent Trilogy (TRG)

The numbers of statistically significant *r*_*s*_ values (*p* <  0.05) of the various MIs to the differences in dose-volumetric parameters between original and reconstructed IMRT plans are shown in Fig. 2. The PI showed statistically significant *r*_*s*_ values most frequently in the TrueBeam STx system (26 cases), whereas the PM showed statistically significant *r*_*s*_ values most frequently in the Trilogy system (37 cases).

## Discussion

In this study, we calculated the various MIs that have been previously suggested to predict IMRT delivery accuracy and then evaluated the performances of the MIs. When reviewing the mean values of MIs comprehensively, they showed inconsistent trends in IMRT plan complexities for the type of linac and treatment sites. This is because that the factors to be considered when calculating the values of MIs were different from one another. The MCS, PA, PI, and PM indices mainly focus on the beam aperture irregularity or the degree of segmentation, whereas the MI_s_, MI_a_, and MI_c, IMRT_ indices mainly focus on the degree of mechanical movements. Nevertheless, for both the TrueBeam STx and Trilogy systems, all MIs except for the MI_s_ and PA indices, indicated that the HN IMRT had the first or second highest complexity. The MI_s_ values seem to have a limitation to calculate the plan complexities properly for various treatment sites since only MLC movements were considered for the MI_s_. The PA values increased as the modulation degree of IMRT increased unlike the existing definition and this was caused by the various target volume sizes for each treatment site in this study. Since the PA regards the use of small-sized apertures as high modulation of a plan, it is not appropriate to evaluate modulation degree of plans with various treatment sites. For example, the target volume sizes of the HN IMRT plans, which were highly modulated, were the largest among all IMRT plans, which resulted in the highest value of PA (the high value of PA means low modulation of a plan by its definition). On the contrary, the target volume sizes of the prostate boost IMRT plans, which were modulated less, were small, which resulted in the lowest value of PA. Therefore, PA increased owing to the target volume size variations not the modulation degree in this study. If we analyzed IMRT plans of a single treatment site with similar target volume sizes, this contradictory tendency to the definition of the PA would not be observed [26, 29]. PA values seem to not provide an appropriate modulation index for predicting IMRT delivery accuracy, through our analysis of IMRT plans with various treatment sites.

For TrueBeam STx, every gamma passing rate with both the MapCHECK2 and ArcCHECK indicated that HN IMRT plans had the lowest gamma passing rates for all gamma criteria. For Trilogy, every gamma passing rate with both the MapCHECK2 and ArcCHECK indicated that prostate boost plans had the lowest gamma passing rates for all gamma criteria, showing the inconsistency with the tendencies in the values of MIs. It was indicated that high-dose gradients of the field edge with low modulation and small field sizes increased the measurement uncertainty [30, 31] and it seems to decrease the gamma passing rates. This may be a factor in lowering correlations between MIs and gamma passing rates. Even for identical IMRT plan deliveries under identical gamma parameter settings, we acquired different gamma passing rates between the MapCHECK2 and ArcCHECK dosimeters, similar the findings of a previous study [15, 31]. Because the MapCHECK2 dosimeter has a high angular dependency, the gamma passing rates become inaccurate when the weight of delivered MUs increase at gantry angles near 90° or 270°. Jin et al. demonstrated that the MapCHECK2 measured doses near a gantry angle of 90° were inaccurate, and the inaccuracy increased as the photon beam energy decreased (30.6, 8.9, and 2.2% at gantry angles of 90° ± 10° for photon beam energies of 6, 10, and 15 MV, respectively) [30, 31]. Similarly, differences in the gamma passing rates between two dosimeters were large for lung SABR of the TrueBeam STx system, which used fields close to 90° or 270°. On the other hand, small differences in gamma passing rates between two dosimeters were observed for spine SABR and liver SABR of the TrueBeam STx and liver IMRT plans of Trilogy, which used fields close to 0° and 180°. The lower gamma passing rates of the MapCHECK2 dosimeter, compared to those of the ArcCHECK dosimeter, were due to dosimeter characteristics.

The DynaLog file is a record of actual motor values with an update rate of 50 ms. The motor values are converted to MLC position values using a conversion table (mlctable.txt). The Trajectory file is a record of the direct MLC position values, with an update rate of 20 ms [30]. The information of the Trajectory file is more accurate than that of the DynaLog file, owing to the short record time and direct-record method [21, 32–34]. With respect to HN IMRT plans, the Trilogy MLC errors were much larger than those of the TrueBeam STx system, but the gamma passing rates of the TrueBeam STx system were smaller and as low as 11.5% of those of the Trilogy system, which were contradictory results to each other. Therefore, a direct absolute comparison between the DynaLog and Trajectory files seems inappropriate, since the recording systems differ. At the very least, we could identify the degree of IMRT plan modulation for various treatment sites within a single linac system.

For TrueBeam STx with the MapCHECK2, the PI showed the highest *r*_*s*_ value for gamma passing rates with 2%/2 mm, 2%/1 mm and 1%/2 mm (*r*_*s*_ = − 0.591 with *p* <  0.001, *r*_*s*_ = − 0.550 with *p* <  0.001 and *r*_*s*_ = − 0.474 with *p* <  0.001, respectively). For the ArcCHECK measurements, the *r*_*s*_ values of the PI were − 0.545 and − 0.677 for gamma passing rates with 2%/2 mm and 1%/2 mm (all with *p* <  0.001), showing the second highest correlations. For the Trilogy system with the MapCHECK2, PI showed the second highest *r*_*s*_ values for every passing rate gamma criterion (all with *p* <  0.001). For ArcCHECK measurements, the moderately strong correlations were observed between PI and the gamma passing rates with 2%/2 mm and 2%/1 mm (*r*_*s*_ = − 0.581 with *p* <  0.001 and *r*_*s*_ = − 0.663 with *p* <  0.001). By reviewing the results of the correlation analysis, PI mostly showed the first or second strongest correlations with every gamma passing rate, regardless of gamma criteria with the MapCHECK2 dosimeter, in both TrueBeam STx and Trilogy results (all with *p* <  0.001). Although the PI did not show the best performance with the ArcCHECK measurements, it showed moderately strong correlations with the gamma passing rates from the ArcCHECK measurements.

In the present study, the first attempt was made to evaluate performance of the previously developed MIs for IMRT by analyzing correlations of MIs with various plan delivery accuracy measures. By utilizing a large number of IMRT plans, various treatment sites, multiple photon energies and linac types, and multiple dosimeters, the correlations in this study were not as high as those found in earlier studies [3, 14, 16, 31]. Among various modulation indices, the PI showed moderately strong correlations with every plan delivery accuracy measure. Therefore, the PI seems to be used to verify IMRT plan delivery accuracy at the planning level in the clinical setting. Furthermore, it has a potential to be integrated in the treatment planning system (TPS) to generate IMRT plans with high delivery accuracy. It is expected to reduce resources in the clinical settings.

Although this study was expanded to comprehensively examine the performance of MIs as predictors of IMRT delivery accuracy by utilizing various treatment sites, photon beam energies, and dosimeters, the results were limited to only Varian linacs. This is a limitation of the present study. To acquire more comprehensive results, further study will be conducted by utilizing various types of linac from various manufacturers in the future.

## Conclusions

The PI value showed best performance, among the modulation indices that were evaluated in this study, as a predictor of IMRT plan delivery accuracy showing strong correlations with various measures of IMRT delivery accuracy. The PI value could support the verification of IMRT plan delivery accuracies before patient treatment and reduce resource consumption in the clinic, as it can be calculated at the planning level.

## Additional file

Additional file 1: **Table S1.** Summary of intensity modulated radiation therapy plan information. **Table S2.** Modulation indices for intensity modulated radiation therapy plans. (DOCX 27 kb)

## Abbreviations

AAA: Anisotropic analytic algorithm
DVO: Dose volume optimizer
FFF: Flattening filter free
HN: Head and neck
IMRT: Intensity-modulated radiation therapy
IRB: Institutional review board
MCS: Modulation complexity score
MI: Modulation index
MLC: Multi-leaf collimator
MU: Monitor unit
OAR: Organ at risk
PA: Plan-averaged beam area
PI: Plan-averaged beam irregularity
PM: Plan-averaged beam modulation
QA: Quality assurance
*r*_*s*_: Spearman’s rho
SABR: Stereotactic ablative radiotherapy
SIB: Simultaneous integrated boost
TPS: Treatment planning system
VMAT: Volumetric modulated arc therapy
