# Supplementary material for: Assessment of the modulation degrees of intensity-modulated radiation therapy plans
Source: Radiat Oncol. 2018 Dec 13;13:244. doi: 10.1186/s13014-018-1193-9 (PMC6293636; doi:10.1186/s13014-018-1193-9)
Supplement: Supplementary file 1 — Table S1. Summary of intensity modulated radiation therapy plan information. Table S2. Modulation indices for intensity modulated radiation therapy plans. (DOCX 27 kb) [file 13014_2018_1193_MOESM1_ESM.docx]

Table S1. Summary of intensity modulated radiation therapy plan information

| Treatment site | *N* | Photon energy | Prescription dose (Gy) | Fraction number | Collimator  angles | Gantry angles |
| --- | --- | --- | --- | --- | --- | --- |
| TrueBeam STx | | | | | | |
| Lung SABR | 20 | 6 MV FFF | 60 | 4 | 30 | Various gantry angles^*^ |
| Spine SABR | 20 | 10 MV FFF | 16 | 1 | 30 | 165°, 140°, 120°, 100°, 85°, 45°, 20°, 205°, 220°, 240°, 260°, 285°, 325°, 340°, 0° |
| Liver SABR | 20 | 10 MV FFF | 42 | 3 | 30 | Various gantry angles |
| Brain | 20 | 6 MV | 30 | 10 | 30 | Various gantry angles |
| Head and neck | 20 | 6 MV | 67.5, 54, 48  (for PTV1, PTV2, PTV3, respectively) | 30 | 5 or 10 | 160°, 100°, 60°, 40°, 320°, 300°, 260°, 200° |
| Trilogy | | | | | | |
| Head and neck | 40 | 6 MV | 67.5, 54, 48  (for PTV1, PTV2, PTV3, respectively) | 30 | 5 or 10 | 160°, 100°, 60°, 40°, 320°, 300°, 260°, 200° |
| Prostate primary | 21 | 15 MV | 50.4 | 28 | 30 | 160°, 100°, 60°, 40°, 320°, 300°, 260°, 200° |
| Prostate boost | 21 | 15 MV | 30.6 | 17 | 30 | 165°, 95°, 65°, 30°, 330°, 295°, 265°, 195° |
| Liver | 11 | 15 MV | 50 | 20 | 30 | 160°, 100°, 60°, 40°, 320°, 300°, 260°, 200° |
| Spine | 9 | 15 MV | 30 | 10 | 30 | 160°, 100°, 60°, 40°, 320°, 300°, 260°, 200° |

Note: H&N = head and neck, PTV = planning target volume, PP = primary plan, BP = boost plan, SABR = stereotactic ablative radiotherapy, FFF = flattening filter free

^*^For brain intensity modulated radiation therapy (IMRT), liver stereotactic ablative radiotherapy (SABR), and lung SABR plans, either coplanar or non-coplanar fields with various gantry angles were used, depending on the target position, target size, and positional relationship between the target volume and organs at risk (OARs).

Table S2. Modulation indices for intensity modulated radiation therapy plans

| MI_s_ | $z_{speed}\left( f \right)=\left( \frac{1}{N_{seg}-1} \right)\cdot\sum_{i=1}^{N_{seg}} N_{i}\left( f;MLC {speed}_{i}>f\sigma_{MLC speed} \right)$  $\mathrm{Individual}\mathrm{MI}_{s}=\int_{0}^{k} z_{speed}\left( f \right) df$  $\mathrm{MI}_{s}=\frac{1}{N_{seg}}\cdot\sum_{n=1}^{120} individual \mathrm{MI}_{s n}$ | *f* = 0.01, 0.02…2  *N_seg_* = the total number of segments  *σ_MLC speed_* = standard deviation of *MLC speed_i_*  *k* = 0.2, 0.5, 1 and 2 |
| --- | --- | --- |
| MI_a_ | $z_{accel}\left( f \right)=\left( \frac{1}{N_{seg}-2} \right)\cdot\sum_{i=1}^{N_{seg}} N_{i}\left( f;MLC {speed}_{i}>f\sigma_{MLC speed} or MLC {accel}_{i}>\alpha f\sigma_{MLC accel} \right)$  $\mathrm{Individual}\mathrm{MI}_{a}=\int_{0}^{k} z_{accel}\left( f \right) df$  $\mathrm{MI}_{a}=\frac{1}{N_{seg}}\cdot\sum_{n=1}^{120} individual \mathrm{MI}_{a n}$ | *f* = 0.01, 0.02…2  *N_seg_* = the total number of segments  *σ_MLC speed_* = standard deviation of *MLC speed_i_*  *σ_MLC accel_* = standard deviation of *MLC accel_i_*  *α* = weighting factor for the acceleration  *k* = 0.2, 0.5, 1 and 2 |
| MI_c, IMRT_ | $z_{comp}\left( f \right)=\left( \frac{1}{N_{seg}-2} \right)\cdot\sum_{i=1}^{N_{seg}} \left\{ N_{i}\left( f;MLC {speed}_{i}>f\sigma_{MLC speed} or MLC {accel}_{i}>\alpha f\sigma_{MLC accel} \right)\cdot W_{AI,i} \right\}$  $\mathrm{Individual}\mathrm{MI}_{comp}=\int_{0}^{k} z_{comp}\left( f \right) df$  $\mathrm{MI}_{c,IMRT}=\frac{1}{N_{seg}}\cdot\sum_{n=1}^{120} individual \mathrm{MI}_{comp n}$ | *f* = 0.01, 0.02…2  *N_seg_* = the total number of segments  *σ_MLC speed_* = standard deviation of *MLC speed_i_*  *σ_MLC accel_* = standard deviation of *MLC accel_i_*  *α* = weighting factor for the acceleration  *W_AI_* = weighting factor for small and irregular shaped beam apertures  *k* = 0.2, 0.5, 1 and 2 |
| MCS | ${LSV}_{segment}=\left( \frac{\sum_{n=1}^{N-1} \left( {pos}_{max}-\left\vert\left( {pos}_{n}-{pos}_{n+1} \right) \right\vert\right)}{N\times{pos}_{max}} \right)_{leftbank}\times\left( \frac{\sum_{n=1}^{N-1} \left( {pos}_{max}-\left\vert\left( {pos}_{n}-{pos}_{n+1} \right) \right\vert\right)}{N\times{pos}_{max}} \right)_{rightbank}$  ${AAV}_{segment}=\frac{\sum_{a=1}^{A} \left( \left\langle{pos}_{a} \right\rangle_{leftbank}-\left\langle{pos}_{a} \right\rangle_{rightbank} \right)}{\sum_{a=1}^{A} \left( \left\langle max({pos}_{a}) \right\rangle_{leftbank\in beam}-\left\langle max({pos}_{a}) \right\rangle_{rightbank\in beam} \right)}$  $MCS=\frac{1}{N_{seg}}\cdot\sum_{i=1}^{I} \left[ {AAV}_{segment, i}\times{LSV}_{segment, i}\times\frac{{MU}_{segment, i}}{{MU}_{beam}} \right]$ | *pos_max_* = maximum distance between MLC positions for a leaf bank  *N* = the number of open leaves inside the jaw  *N_seg_* = the total number of segments  *pos* = MLC position  *LSV* = leaf sequence variability  *AAV* = aperture area variability  *A* = the number of leaves in the leaf bank  I = the number of segments in the beam |
| PA | $\mathrm{BA}_{i}=\frac{\sum_{j} \left( {MU}_{ij}\cdot{AA}_{ij} \right)}{{MU}_{i}}$  $PA=\frac{\sum_{i} \left( {BA}_{i}\cdot{MU}_{i} \right)}{{MU}_{P}}$ | *AA_ij_* = aperture area for *j*th segment in *i*th beam  *BA* = beam area  *MU_ij_* = MU of segment *AA_ij_*  *MU_i_* = MU of beam  *MU_P_* = total MU in the plan |
| PI | ${AI}_{ij}=\frac{{AP}_{ij}^{2}}{4\pi\cdot{AA}_{ij}}$  $\mathrm{BI}_{i}=\frac{\sum_{j} \left( {MU}_{ij}\cdot{AI}_{ij} \right)}{{MU}_{i}}$  $PI=\frac{\sum_{i} \left( {BI}_{i}\cdot{MU}_{i} \right)}{{MU}_{P}}$ | *AI_ij_* and *AP_ij_* = aperture irregularity and aperture perimeter for *j*th segment in *i*th beam  *BI* = beam irregularity  *MU_ij_* = MU of segment *AA_ij_*  *MU_i_* = MU of beam  *MU_P_* = total MU in the plan |
| PM | $\mathrm{BM}_{i}=1-\frac{\sum_{j} \left( {MU}_{ij}\cdot{AA}_{ij} \right)}{{MU}_{i}\cdot U({AA}_{ij})}$  $PM=\frac{\sum_{i} \left( {BM}_{i}\cdot{MU}_{i} \right)}{{MU}_{P}}$ | *BM* = beam modulation  *U(AA_ij_)* = the union area of all apertures of beam *i*  *MU_ij_* = MU of segment *AA_ij_*  *MU_i_* = MU of beam  *MU_P_* = total MU in the plan |

Note: MI_s_ = modulation index quantifying multi-leaf collimator speeds, MI_a_ = modulation index quantifying multi-leaf collimator accelerations, MI_c, IMRT_ = modulation index quantifying multi-leaf collimator accelerations and field aperture irregularity for intensity modulated radiation therapy, MCS = modulation complexity score, PA = plan-averaged beam area, PI = plan-averaged beam irregularity, PM = plan-averaged beam modulation
